# Acute kidney injury in hospitalized patients with COVID-19 (retrospective study)

**DOI:** 10.1186/s43168-021-00056-z

**Published:** 2021-01-12

**Authors:** Haitham A. Azeem, Hytham Abdallah, Mohamad M. Abdelnaser

**Affiliations:** 1 Internal Medicine Department, Faculty of Medicine, Al-Azhar University (Assiut), Al-Azhar University Square, King Faysal, Assiut, 71524 Egypt; 2 Chest Diseases Department, Faculty of Medicine, Al-Azhar University (Assiut), Al-Azhar University Square, King Faysal, Assiut, 71524 Egypt

## Abstract

**Background:**

The World Health Organization (WHO) has named the virus as 2019 novel coronavirus on January 12, 2020, and has declared a public health emergency globally on January 30, 2020. The epidemic started in Wuhan, China, in December of 2019 and quickly spread to over 200 countries. COVID-19 can cause multiple organ injuries (e.g., kidney, heart, blood, and nervous system). Among them, acute kidney injury (AKI) is a critical complication due to its high incidence and mortality rate. So, it is essential to evaluate AKI in COVID-19 patients during this pandemic state. The aim of this work is to detect the occurrence of AKI in hospitalized COVID-19 patients. So, a retrospective study was conducted on hospitalized adult patients > 18 years old with confirmed SARS-CoV-2 infection admitted to the Abo Teeg Hospital at Assiut City, Egypt, from May 1, 2020, to July 1, 2020. All data were collected from medical records, patients’ follow-up, and charts. Data were verified, coded by the researcher, and analyzed using IBM-SPSS 21.0.

**Results:**

Eighty-six COVID-19 patients were admitted to Abo Teeg Hospital in Assiut City, Egypt, between May and July 2020. Thirty-eight patients (33%) were of the male gender. Mean age was 58.07 ± 17.9, and 61 patients developed AKI. 32.8% of the AKI group were a stage I severity (increase in serum creatinine by 0.3 mg/dl within 48 h), 21.3% of them presented by stage II (2–2.9 times increase in serum creatinine), and 45.9% were in stage III (3 times or more increase in serum creatinine). The overall hospital mortality for the patients admitted to ICU with AKI was 6.7% (11/61), compared to 1% (4/25) in those without AKI.

**Conclusion:**

Hospitalized patients with COVID-19 had a higher risk of AKI, and we recommended that those patients should be evaluated after discharge for the development of CKD.

## Background

The 2019 novel coronavirus disease (COVID-19) is a newly defined serious infectious disease caused by the SARS-CoV-2 virus. The World Health Organization (WHO) has named the virus as 2019 novel coronavirus (2019-nCoV) on January 12, 2020, and has declared a public health emergency globally on January 30, 2020 (https://www.who.int/news-room/detail/30-01-2020-statement-on-the-second-meeting-of-the-international-health-regulations-(2005)-emergency-committee-regarding-the-outbreak-of-novel-coronavirus-(2019-ncov)). There was no determination on the animal species carrying the nCoV. The preliminary studies showed that the nCoV is closely related to the coronavirus isolated from bats, postulating the theory of possible transmission from bats to humans [1].

The epidemic started in Wuhan, China, in December of 2019 and quickly spread to over 200 countries. It has affected 4,258,666 people, with 294,190 deaths worldwide by May 15, 2020. COVID-19 is characterized by acute respiratory disease, with 80% of patients presenting mild flu-like symptoms; however, 20% of patients may have a severe or critical clinical presentation, which likely causes multiple organ injuries (e.g., kidney, heart, blood, and nervous system). Among them, acute kidney injury (AKI) is a critical complication due to its high incidence and mortality rate [2]. Multiple mechanisms are involved in COVID-19-associated AKI, ranging from direct viral infection of the kidney and secondary inflammation to complement activation and microthrombosis [3]. AKI in COVID-19 infection could be resulting from the synergistic effect of virus-induced direct cytotropic effect and cytokine-induced systemic inflammatory response [4].

## Methods

A retrospective observational study was conducted on adult patients admitted to the Abo Teeg Hospital, Assiut, Egypt, with laboratory and radiologically confirmed SARS-CoV-2 infection between May 1, 2020, and July 1, 2020, under the supervision of our medical staff of Al-Azhar University Hospital, Assiut Branch. Patients with known end-stage kidney disease (ESKD) before admission and patients who were hospitalized for < 48 h were excluded.

### Definition of AKI

KDIGO (Kidney Disease Improving Global Outcomes) criteria were used to define AKI according to both urinary output and serum creatinine as follows: stage I, an increase in serum creatinine by 0.3 mg/dl within 48 h or 1.5–1.9 times increase in serum creatinine from baseline or urinary output < 0.5 ml/kg/h for 6–12 h within 7 days; stage II, 2.9 times increase in serum creatinine or urinary output < 0.5 ml/kg/h for ≥ 12 h within 7 days; and stage III, 3 times or more increase in serum creatinine or to ≥ 4.0 mg/dl or urinary output < 0.3 ml/kg/h for ≥ 24 h or anuria for ≥ 12 h within 7 days. Baseline creatinine was defined as the best value in the 3 preceding months, if unavailable as the lowest value during the hospital stay or was back-calculated based on a glomerular filtration rate of 60 ml/min/1.73 m^2^ with MDRD (Modification of Diet in Renal Disease) equation in patients without known chronic kidney disease [5].

### Data collection

All data were collected from medical records, patients’ follow-up, and charts. Baseline patients’ characteristics were collected (Table 1), including demographics and comorbidities before hospital admission, and clinical, laboratory characteristics, and outcomes data were obtained.

**Table 1:** Baseline Patients characteristics of the study groups

Parameter	All (***n*** = 86)	AKI (***n*** = 61)	Non-AKI (***n*** = 25)	***P*** value
**Age, years**	58.07 ± 17.9	55.28 ± 17.8	65.48 ± 16.5	0.017*
**Sex (male/female)**	38/48	31/30	7/18	0.044**
**Onset of symptoms**				
• **Acute**	7 (8.1%)	5 (8.2%)	2 (8%)	0.891**
• **Gradual**	32 (37.2%)	23 (37.7%)	9 (36%)	
• **Sudden**	47 (54.7%)	33 (45.1%)	14 (56%)	
**Comorbidity**				
• **IHD**	14 (17.1%)	14 (23%)	0 (0%)	0.010**
• **DM**	22 (26.8%)	15 (24.6%)	7 (33.3%)	0.305**
• **HTN**	36 (41.9%)	28 (45.9%)	8 (62%)	0.047**
• **CKD**	8 (9.3%)	8 (13.1%)	0 (0%)	0.045**
• **Liver disease**	6 (7%)	5 (8.2%)	1 (4%)	0.134**
• **Others**	22 (25.6%)	17 (27.9%)	5 (20%)	0.041**

*Independent *t* test was used to compare the means among groups

**Chi-square analysis was used to compare the frequency among groups

### Statistical analysis

Data were verified, coded by the researcher, and analyzed using IBM-SPSS 21.0 (IBM-SPSS Inc., Chicago, IL, USA). For descriptive statistics, means, standard deviations, medians, and ranges were calculated. For the test of significances, chi-square/Fisher’s exact test was calculated to compare the frequencies among groups. For continuous variables, independent *t* test analysis was carried out to compare the means of normally distributed data, while the Mann-Whitney *U* test was calculated to test the median differences of the data that do not follow a normal distribution. The Kaplan-Meier curve was used to estimate the median survival time. The log-rank test was used to compare survival curves between the categories of the explanatory variables. A significant *P* value was considered when it is less than 0.05 [6].

## Results

**Fig. 1:**
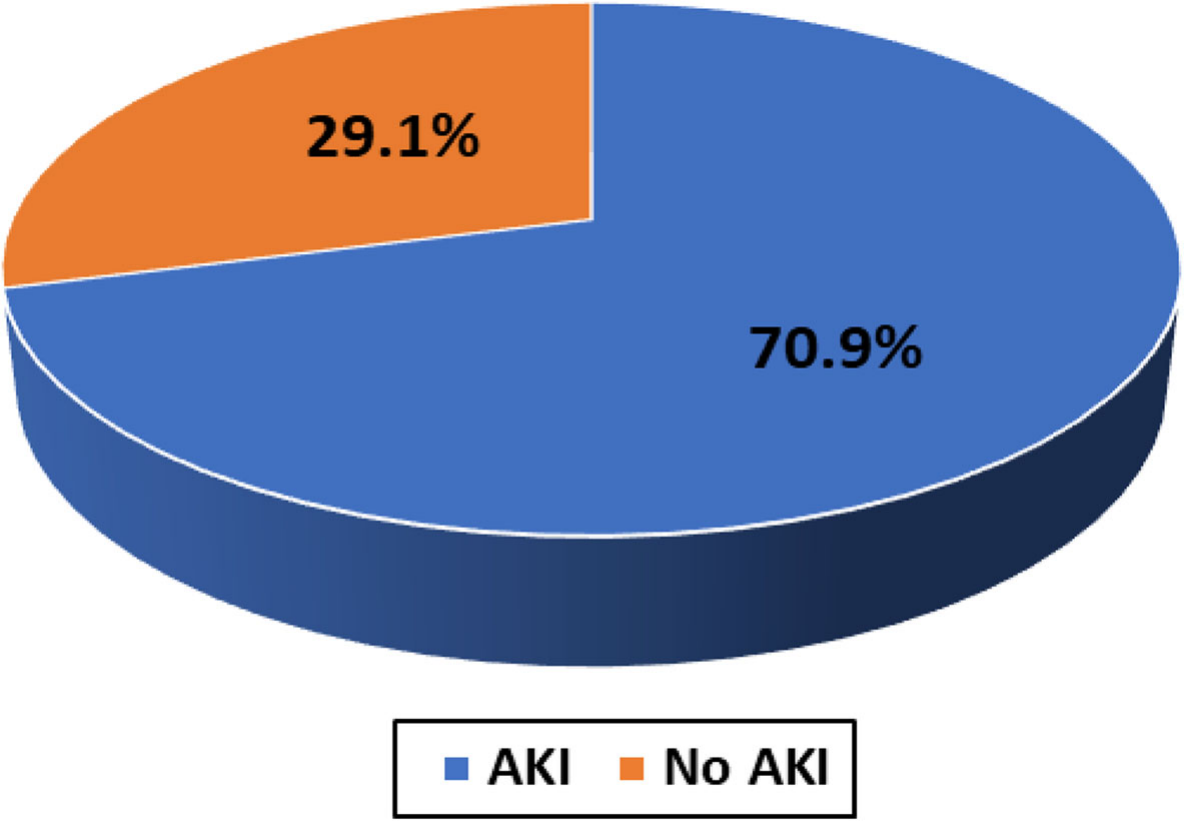
Frequency of AKI among the studied COVID-19 patients cohort

**Fig. 2:**
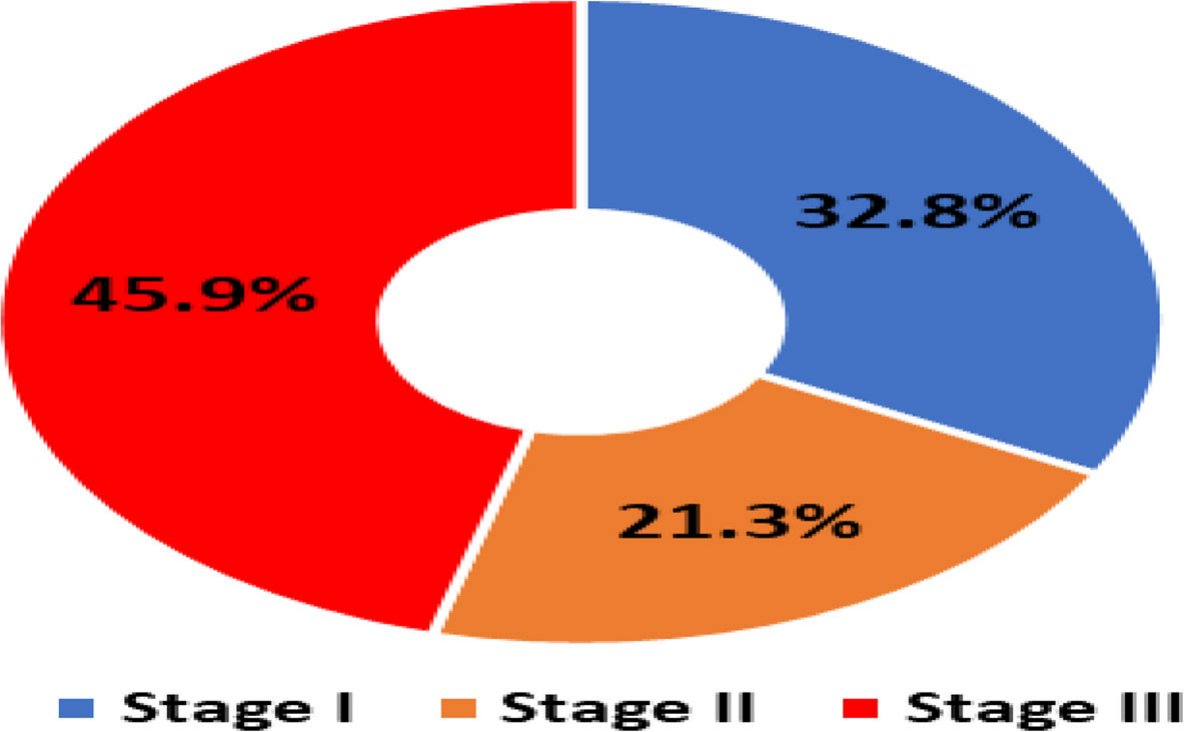
Severity of AKI among the studied COVID-19 patients cohort

**Fig. 3:**
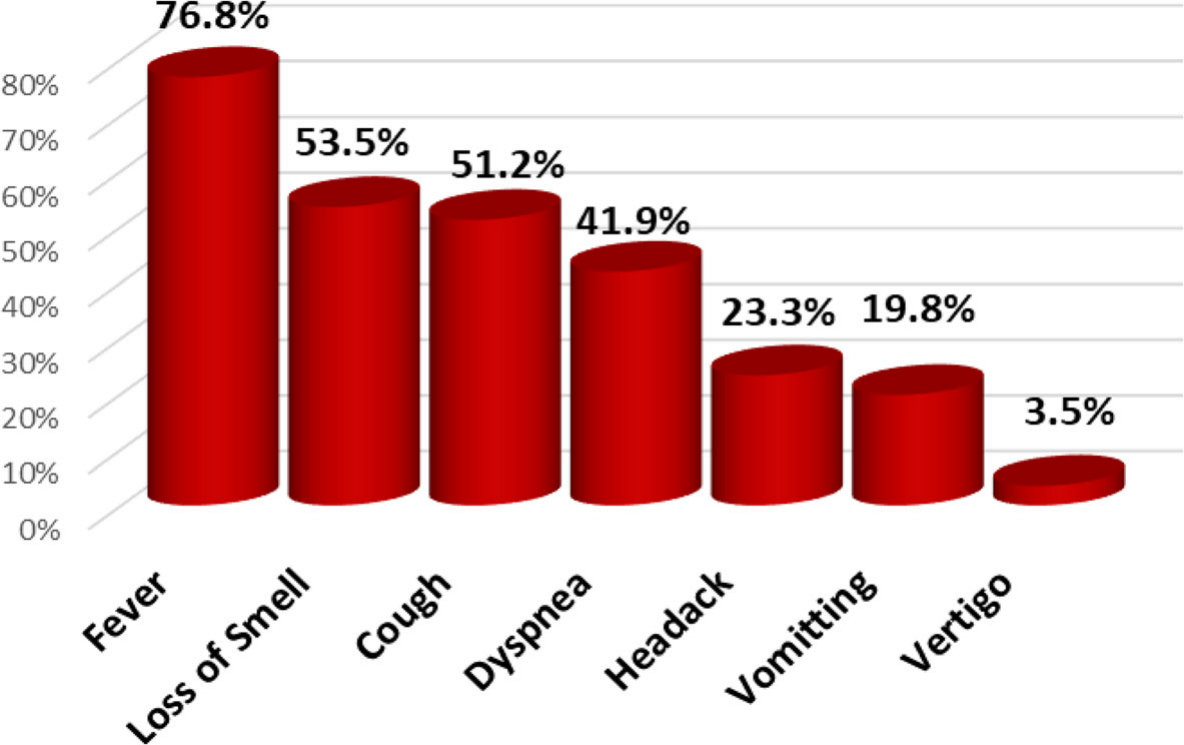
COVID-19 symptoms frequency among the studied patients cohort

**Fig. 4:**
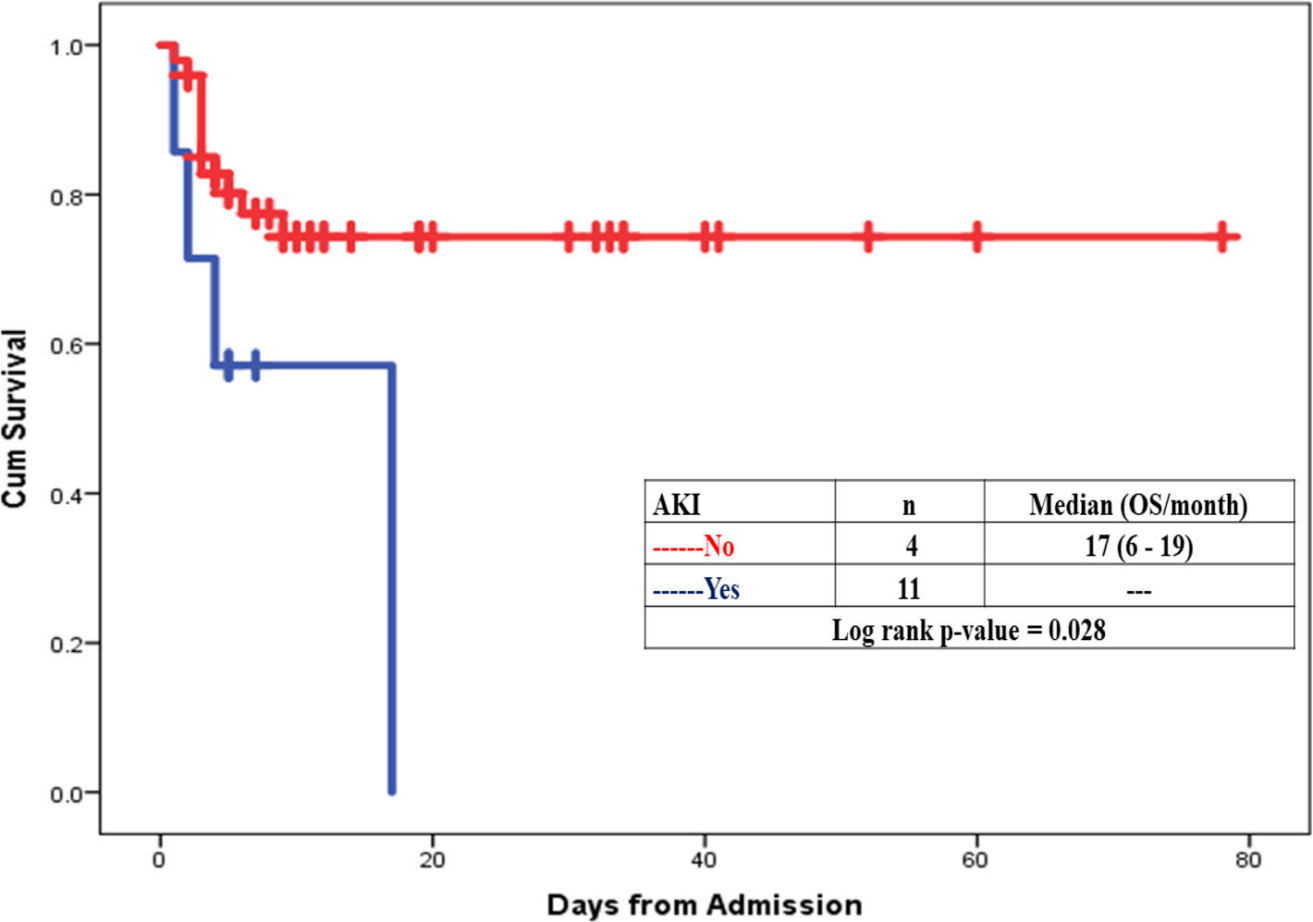
Survival probability is lower in patients with AKI. The Kaplan-Meier survival curves for patients with and without AKI. Red line indicates patients without AKI, and blue line is for those with AKI. AKI patients were censored at 17 days. Patients who were discharged alive before 17 days were treated as still at risk and not censored at discharge

Overall, 86 COVID-19 patients were admitted in Abo Teeg Hospital in Assiut City, Egypt, between May and July 2020 which were included in this study. Mean age was 58.07 ± 17.9, and 38 patients (33%) were of the male gender. 8.2% of those patients developed acute COVID-19 symptoms (8% in the non-AKI group), 37.7% developed gradual onset (36% in the non-AKI group), and lastly 45.1% developed symptoms suddenly (56% in the non-AKI group). Within 7 days of admission, 61 patients developed AKI and meet the KDIGO criteria. The AKI group had a higher grade of fever than the non-AKI group with no significant differences in other symptoms such as cough, dyspnea, and loss of smell (Table 2). The AKI group had higher comorbidity than the non-AKI group. Associated comorbidities such as hypertension (*n* = 36, 41.9%), diabetes mellitus (*n* = 22, 26.8%), and chronic kidney disease (*n* = 8, 9.3%) were observed, in addition to ischemic heart disease (*n* = 14, 17.1%) and chronic liver disease (*n* = 6, 7%). As regards vital signs, the AKI group had a lower PO_2_ (80.5 (28–224)) and SO_2_ (95 (46–100)) than the non-AKI group with no significant differences as regards blood pressure, blood PH, and PCO_2_ (Table 3). On the other hand, the AKI group had lower values of Hbg (11.5 (5–15)), lymphocyte (13 (3–22)), and platelets (207 (110–727)) with significant differences than the non-AKI group. The AKI group had higher serum creatinine with median 0.7 (0.02–6.25) and BUN (17 (9–176)) than the non-AKI group in which the results are 0.2 (0.01–0.1) and 7 (1–85), respectively. The AKI group shows higher INR (1.04 (0.9–4.6)) and lower albumin (3.4 (0.2–5.1)) comparing with the non-AKI group. Lastly, the AKI group showed higher levels of C-reactive protein, serum ferritin, and serum D-dimer than the non-AKI group (46 vs 38, 659 vs 409, and 6789 vs 2345, respectively). 32.8% of the AKI group show stage I severity (increase in serum creatinine by 0.3 mg/dl within 48 h), 21.3% of them presented by stage II (2–2.9 times increase in serum creatinine), and 45.9% were in stage III (3 times or more increase in serum creatinine). For patients admitted to ICU with AKI (11/61) (6.7%), the median time from ICU admission to documented peak AKI was 4 days. For patients who received renal replacement therapy (RRT), the median time to commencing RRT was 5 days (IQR 9 days) after admission to the intensive care unit and the median duration of RRT was 9 days (IQR 11 days). The overall hospital mortality for the patients admitted to ICU with AKI was 6.7% (11/61), compared to 1% (4/25) in those without AKI. Survival probability is lower in patients with AKI. The Kaplan-Meier survival curves for patients with and without AKI were shown. AKI patients were censored at 17 days. Patients who were discharged alive before 17 days were treated as still at risk and not censored at discharge (Figs. 1, 2, 3, and 4).

**Table 2:** COVID-19 symptoms and signs of the studied groups

Parameter	All (***n*** = 86)	AKI (***n*** = 61)	Non-AKI (***n*** = 25)	***P*** value*
**Clinical manifestations**				
• **Fever**	66 (76.8%)	50 (82%)	16 (64%)	**0.036**
• **Headache**	20 (23.3%)	15 (24.6%)	5 (20%)	0.239
• **Vomiting**	17 (19.8%)	12 (19.7%)	5 (20%)	0.480
• **Vertigo**	3 (3.5%)	3 (4.9%)	0 (0%)	0.152
• **Cough**	44 (51.2%)	34 (55.7%)	10 (40%)	0.098
• **Dyspnea**	36 (41.9%)	24 (39.3%)	12 (48%)	0.308
• **Loss of smell**	46 (43.5%)	29 (47.5%)	17 (68%)	0.086

*Chi-square analysis was used to compare the frequency among groups

**Table 3:** Vital signs and lab. differences among the studied COVID-19 patients

Parameter	All (***n*** = 86)	AKI (***n*** = 61)	Non-AKI (***n*** = 25)	***P*** value*
**Vital signs**				
• **SBP (mmHG)**	120 (70–210)	140 (100–210)	120 (70–170)	0.089
• **DBP (mmHG)**	80 (50–110)	80 (50–110)	80 (60–100)	0.362
• **PH**	7.41 (7.3–7.6)	7.43 (7.3–7.5)	7.38 (7.5–7.6)	0.658
• **PCO**_**2**_	31 (20–62)	34 (25–56)	26 (20–62)	0.112
• **PO**_**2**_	87 (28–230)	80.5 (28–224)	91 (85–230)	**0.044**
• **SO**_**2**_	98 (46–100)	95 (46–100)	99 (80–100)	**0.041**
**Laboratory findings**				
• **WBCs (10**^**3**^**/μl)**	10 (3.5–27)	10 (3.5–27)	9.5 (8–15)	0.684
• **Lymphocyte (%)**	14 (2.5–55)	13 (3–22)	15 (2.5–50)	**0.041**
• **Hgb (g/dl)**	12 (5–15)	11.5 (5–15)	12.5 (11–14)	**0.046**
• **Platelet (10**^**3**^**/l)**	266 (110–727)	207 (110–727)	266 (115–624)	**0.025**
• **Neutrophil (%)**	75 (22–94)	78 (28–94)	72 (28–86)	0.215
• **BUN (mg/dl)**	11 (1–176)	17 (9–176)	7 (1–85)	**0.005**
• **S. creatinine**	0.5 (0.01–6.25)	0.7 (0.02–6.25)	0.2 (0.01–0.1)	**0.011**
• **AST (U/l)**	31 (16–423)	35 (27–423)	29 (16–114)	0.098
• **ALT (U/l)**	31 (5–312)	28 (5–312)	34 (5–84)	**0.033**
• **T. bilirubin (mg/dl)**	8 (0.5–45)	10 (2–45)	4 (0.5–20)	**0.045**
• **Albumin (g/dl)**	3.7 (0.1–5.1)	3.4 (0.2–5.1)	3.9 (0.1–4.5)	**0.016**
• **PT**	15 (9–45)	13 (11–45)	14 (9–18)	0.146
• **PC%**	93 (18–124)	92 (18–124)	95 (75–100)	0.245
• **INR**	1.01 (0.7–4.6)	1.04 (0.9–4.6)	0.82 (0.7–1.1)	**0.040**
• **CRP (IU/ml)**	44 (6–99)	46 (6–99)	38 (10–83)	**0.049**
• **Ferritin (ng/ml)**	612 (76–6543)	659 (76–6543)	409 (125–903)	**0.003**
• **D-dimer (ng/ml)**	5946 (98–13,470)	6789 (356–13,470)	2345 (98–9876)	**0.001**

*Mann-Whitney *U* test was used to compare the medians among groups

## Discussion

AKI is a common complication among patients hospitalized for a wide range of diagnoses. The etiology of AKI in COVID-19 cases has not been fully elucidated. The close temporal relationship between AKI and respiratory failure occurrence is somewhat suggestive of ischemic acute tubular necrosis. The prothrombotic state that has been observed among patients with COVID-19 suggests other renal pathogenic factors [7]. Clinically, the incidence of acute kidney injury in COVID-19 varied from 0 to 66% in different centers [7]. For evaluation of AKI in hospitalized patients with COVID-19, a retrospective observational study was conducted, which included adult patients with laboratory and radiologically confirmed SARS-CoV-2 infection admitted to the Abo Teeg Isolation Hospital, Assiut, Egypt, under supervision of our medical staff of Al-Azhar University Hospital, Assiut Branch. All patients above 18 years were hospitalized from May 1, 2020, to July 1, 2020, and all data related to those patients were collected from medical records and patients’ charts. Vital data and laboratory findings were demonestrated in Table 3. Within 3–14 days (7 days average) of admission, 61 (70.9%) patients developed AKI and meet the KDIGO criteria   (Fig. 1). Twenty patients (32.8%) of the AKI group show stage I severity, 13 patients (21.1%) of the AKI group show stage II severity, and 28 patients (45.9%) of the AKI group show stage III severity (Fig. 2). Comparing with a study performed by Jamie et al. on 5449 COVID-19 patients, 1993 (36.6%) of them developed AKI during their hospitalization, where a higher rate of stage I (46.5%) was observed and the lowest rate of stage II detected in 31.1% and lastly 22.4% show stage III severity [8]. This is a higher rate than has been reported previously from China and other areas, from smaller studies, and including various stages of the disease. For example, the rate of AKI reported has ranged from 0.5 to 29%. In particular, Cheng et al. [9] reported from Wuhan, China, a rate of AKI of only 5.1% of 701 patients. While we cannot completely explain this difference, it must be noted that significantly lower rates of comorbidities such as diabetes and hypertension were reported in their patients. A similar study was performed by Lili et al. [10], which was a prospective and observational study on 3993 hospitalized patients with COVID-19, where AKI has occurred in 1835 (46%) patients; 347 (19%) of the patients required hemodialysis. Stages I, II, and III AKI were 39%, 19%, and 42%, respectively. A total of 976 (24%) patients were admitted to intensive care, and 745 (76%) experienced AKI. Independent predictors of severe AKI were CKD, men, and higher serum potassium in those patients at admission [8]. In our study, the AKI group had higher comorbidity than the non-AKI group, where hypertension was observed in 28 patients (45.9%), DM in 15 patients (24.6%), IHD in 14 patients (23%), CKD in 8 patients (13.1%), and lastly chronic liver disease in 5 patients (8.2%) as shown in ( Table 1), which was in agreement with Lili et al.’s study which observed that patients who developed incident AKI were older and were more likely to have hypertension, congestive heart failure, diabetes mellitus, and CKD [8]. Our study shows that the AKI group had a higher clinical presentation by fever, cough, dyspnea, and loss of smell than the non-AKI group as showen in (Table 2 & Fig. 3). Also, the AKI group had higher comorbidity than the non-AKI group, where hypertension was observed in 28 patients (45.9%), DM in 15 patients (24.6%), IHD in 14 patients (23%), CKD in 8 patients (13.1%), and lastly chronic liver disease in 5 patients (8.2%), (Table 1). When comparing our study with another one performed to evaluate the risk of serious adverse outcomes in COVID-19 patients according to the number and type of comorbidities, Cheng et al. [9] have analyzed 1590 laboratory-confirmed hospitalized patients in 31 provinces/autonomous regions/provincial municipalities from mainland China; 25.1% (399/1590) was reported to have at least one comorbidity. 8.2% (131/1590) of patients reached the composite endpoints, which consisted of admission to intensive care unit, or invasive ventilation, or death, and 8.2% (130/1590) had two or more comorbidities. The prevalence of CKD was 1.3% (21/1590). It is the lowest one compared with hypertension (269; 16.9%), diabetes (130; 8.2%), other cardiovascular diseases (59; 3.7%), cerebrovascular diseases (30; 1.9%), hepatitis B infections (28; 1.8%), or chronic obstructive pulmonary disease (COPD) (24; 1.5%). We noted that, from this study and others, hypertension and diabetes are common comorbidities with COVID-19 patients but the occurrence of AKI in those patients is usually precipitated by COVID-19 where those patients were with near-normal renal profile despite the comorbidities before admission.

The overall hospital mortality for the patients admitted to ICU with AKI was 6.7% (11/61), compared to 1% (4/25) in those without AKI. Survival probability is lower in patients with AKI. The Kaplan-Meier survival curves for patients with and without AKI were shown in Fig. 4. AKI patients were censored at 17 days. Patients who were discharged alive before 17 days were treated as still at risk and not censored at discharge. Our finding is in agreement with the finding of Intensive Care National Audit and Research Centre, which demonstrate that patients with AKI had more severe illness generally, required invasive mechanical ventilation, and had higher illness severity scores, persistent lymphopenia, and vasopressor support, suggesting that AKI is a marker of disease severity, where the study was performed between May and July 2020; a total of 81 critically ill COVID-19 patients were admitted to the intensive care unit. Mortality rates for all the AKI groups, stage I, stage II, and the RRT group are 33%, 28.5%, and 33%, respectively. This is lower than the 60% national mortality in patients receiving RRT reported in the UK ICNARC outcome dataset [11].

The present study has limitations. First, because this is a retrospective and observational study, we cannot make a causal relationship between exposures and AKI where renal biopsy may be needed to prove the direct link between AKI and COVID-19 and explain such role of comorbidities. Second, we cannot generalize our findings in the outpatient AKI settings because non-hospitalized patients were not part of the present study.

## Conclusion

This study concluded that acute kidney injury is a serious feature in critically ill hospitalized patients with COVID-19. It is more common in patients with comorbidities such as hypertension and diabetes in addition to the development of AKI which is associated with increased severity of illness, prolonged duration of hospitalization, and increased mortality. So, we recommended that hospitalized patients with COVID-19 should be evaluated for development of acute renal injury and should be regularly tested for renal functions after discharge to monitor those patients for the progression to CKD.

## Data Availability

Not applicable.

## Abbreviations

WHO: The World Health Organization
AKI: Acute kidney injury
KDIGO: Kidney Disease Improving Global Outcomes
ESKD: End-stage kidney disease
